# The Absence of Obstructive Sleep Apnea May Protect against Non-Alcoholic Fatty Liver in Patients Undergoing Bariatric Surgery

**DOI:** 10.1371/journal.pone.0062504

**Published:** 2013-05-03

**Authors:** Kathleen E. Corey, Joseph Misdraji, Hui Zheng, Kyle M. Malecki, Jacob Kneeman, Louis Gelrud, Raymond T. Chung

**Affiliations:** 1 Gastrointestinal Unit, Massachusetts General Hospital, Boston, Massachusetts, United States of America; 2 Department of Pathology, Massachusetts General Hospital, Boston, Massachusetts, United States of America; 3 MGH Biostatistics Center, Massachusetts General Hospital, Boston, Massachusetts, United States of America; 4 Harvard Medical School, Boston, Massachusetts, United States of America; 5 Department of Internal Medicine, Bon Secours Richmond Health System, Richmond, Virginia, United States of America; Hospital General Dr. Manuel Gea González, Mexico

## Abstract

**Background:**

Non-alcoholic fatty liver disease (NAFLD) is the most common cause of liver disease worldwide and its progressive form, steatohepatitis, will be the leading indication for liver transplant by 2020. While risk factors for steatohepatitis have been identified, little work has been performed to identify factors protective against NAFLD development.

**Aim:**

This study sought to identify factors predictive of normal liver histology in a bariatric cohort.

**Methods:**

Patients undergoing weight loss surgery with liver biopsies at the time of surgery were included. Patients with other causes of chronic liver disease were excluded.

**Results:**

One hundred fifty-nine patients were included. Forty-nine patients had normal liver histology and 110 patients had NAFLD. Several previously identified factors associated with normal liver histology were found. Black race was the strongest predictor of the absence of NAFLD with an odds ratio (OR) of 6.8, 95% confidence interval (CI) 2.4–18.9. Low HOMA-IR was also associated with normal histology (OR 1.4, 95% CI 1.03–1.9). In contrast, low HDL was associated with a *decreased* chance of normal histology (OR 0.38, 95% CI 0.05–0.83). Interestingly, a novel protective factor, the absence of obstructive sleep apnea (OSA) was strongly associated with normal histology (OR 5.6, 95% CI 2.0–16.1). In multivariate regression controlling for BMI, black race, absence of OSA, low HOMA-IR and low ALT independently predicted normal liver histology with an area under the ROC curve of 0.85.

**Conclusions:**

Our study confirmed several factors associated with normal liver histology, including black race and identified a novel factor, absence of OSA. Further evaluation of these factors will allow for improved understanding of the pathogenesis of NAFLD.

## Introduction

Non-alcoholic fatty liver disease (NAFLD) is the most common cause of liver disease in the United States with a growing prevalence worldwide.[1] NAFLD is associated with an increased risk of mortality from both liver disease and cardiovascular disease.[2], [3] Further, non-alcoholic steatohepatitis (NASH), the progressive form of NAFLD, can lead to the development of cirrhosis and hepatocellular carcinoma and is predicted to be the leading indication for liver transplantation by the year 2020.[4]

With the rising prevalence of NAFLD and an increased appreciation of its health consequences, the merit of NAFLD screening in high risk populations has been raised. Currently, no guidelines for NAFLD screening exist, even among high risk patients with diabetes and obesity.[5] Screening for NAFLD would allow for the early identification of patients with NAFLD but would likely be prohibitive in scope and expense. Extensive research is ongoing to identify patients at highest risk for NASH and advanced fibrosis. In addition to the identification of risk factors for NASH, the identification of factors that predict the *absence* of NAFLD should also aid in restricting screening to highest risk patients. The identification of protective factors against NAFLD will contribute to an understanding of NAFLD pathogenesis. To date, no protective factors against NAFLD have been elucidated.

NAFLD is associated with obesity, and up to 91% of obese adults have some form of fatty liver disease.[6], [7] Importantly, while NAFLD is common in patients with obesity, it is not universal. This has been demonstrated in several studies of patients undergoing weight loss surgery. At many centers, a standard of care liver biopsy is performed at the time of weight loss surgery because of the high prevalence of NAFLD. These biopsies have informed many studies evaluating the prevalence of NAFLD and paired biopsy studies have shown that NASH regresses after bariatric surgery in up to 69.5% of patients.[7] Further, these studies have made note of up to a 22% prevalence of normal liver histology. However, the factors associated with normal liver histology in patients at high risk for NAFLD are unknown.

Patients with NASH have been noted to have high rates of atherogenic dyslipidemia, characterized by low high density lipoprotein (HDL), high triglycerides and an increase in small dense low density lipoproteins (LDL). Recent work from our group has found a high prevalence of elevated non-HDL cholesterol (non-HDL-C) in patients with NASH compared to those with NAFLD.[8] We hypothesized that high levels of HDL and low levels of triglyceride and non-HDL-C levels will be predictive of the absence of NAFLD. We also hypothesized that race is predictive of the likelihood of having NAFLD. Radiographic NAFLD has been shown to have the highest prevalence in Hispanic patients and lowest prevalence in African Americans. Thus, we predict that African American race can be used along with other factors to predict absence of histologic NAFLD. This study sought to evaluate these and other factors to determine their combined ability to predict normal liver histology in patients with obesity undergoing weight loss surgery.

## Materials and Methods

### Ethics Statement

This study was approved by the Partners Human Research Committee. Written informed consent was obtained from all subjects.

### Study design and population

Patients were selected from a cohort undergoing weight loss surgery at Bon Secours Healthcare in Richmond, Virginia, from August 2009 to July 2011. Patients age 18 or older were eligible for participation in this study. Patients selected for weight loss surgery did not undergo adjuvant dietary or medication treatment prior to surgery. Patients with chronic liver disease other than non-alcoholic fatty liver disease, as assessed by viral serologies, autoimmune markers, iron studies and liver biopsy, were excluded from analysis. Patients with excess alcohol use, defined as >2 drinks per day for men and >1 drink per day for women were also excluded from the study.

### Data collection

At their final pre-operative visit, patient demographic and metabolic data were collected including age, gender, race, weight, height, and waist and hip circumference.

Subjects were assessed by their treating physician for the presence of co-morbid diseases including diabetes mellitus, hypertension, obstructive sleep apnea, and dyslipidemia. Diabetes mellitus and OSA was defined by a known prior diagnosis of diabetes or OSA in the medical record. Hypertension was defined by a blood pressure > = 135/85 or undergoing treatment for hypertension. Hyperlipidemia was defined as use of hyperlipidemic medications or a previous diagnosis of hyperlipidemia in the medical record.

Subjects underwent fasting blood samples within 2 weeks of their liver biopsy. Samples were analyzed for liver function tests, insulin level, C-reactive protein, glucose level, lipid panel including total cholesterol, LDL, HDL, and triglycerides. Non-HDL-C was calculated by subtracting HDL cholesterol from total cholesterol levels.

All patients had a needle liver biopsy at the time of surgery. All liver biopsies were read by a single blinded hepatopathologist (JM). Grade of steatosis and degree of lobular inflammation and ballooning were assessed to calculate the NAFLD activity score (NAS), and the NASH CRN histologic scoring system was used to stage fibrosis.[9] NAFLD was characterized by the presence of steatosis score of 1 or greater. Normal histology was characterized by score of 0 for steatosis, lobular inflammation, hepatocyte ballooning and fibrosis as well as the absence of other histologic abnormalities.

### Statistical Analysis

Statistical analysis was performed using SAS software, version V.9.2 (SAS Institute, Cary, NC). Continuous variables were analyzed using a Student's t-Test while categorical variables were analyzed using a Chi square test or Fisher's exact test as appropriate. Univariate analysis was used to identify factors that significantly predicted the absence of NAFLD. Factors found to be significant were then assessed using a multivariate linear regression model. A multivariate logistic regression model was performed to identify significant clinical and metabolic factors that predicted the absence of NAFLD after adjusting for other factors including BMI. A final logistic model was selected and the weighting for the predictive scores were derived from the coefficients from the final logistic model. From this fixed model a receiver operating curve was determined and candidate thresholds for the included variables were compared based on sensitivity and specificity. A score was constructed based on a multivariate logistic regression model predicting the probability for NAFLD. The model includes race, obstructive sleep apnea, HOMA-IR and ALT as predictors of NAFLD.

This study was approved by the Partners Human Research Committee.

## Results

### Study Population Clinical Characteristics

A total of 159 patients were included in the study. Characteristics of the patients are described in Table 1. Forty-nine patients (30.8%) had normal liver histology, while 110 patients met criteria for NAFLD. The mean age of patients with normal liver histology was 40.3 years, whereas that of patients with NAFLD was significantly higher at 47.8 years (p = 0.0007). Gender and body mass index were not significantly different between the two groups. Black patients accounted for 57.5% of the normal group but only 19.3% of the NAFLD group (p<0.0001). Among the NAFLD patients, 31.0% (n = 34) had a NAS score of 3–4 and 25.5% (n = 28) had a NAS score > = 5. The majority of NAFLD patients had stage 0 or stage 1 fibrosis (n = 97, 88%) and grade 1 steatosis (n = 61. 55.5%). (Table 2) No difference was seen in the proportion of patients with coronary artery disease or tobacco use between the two groups. Patients with normal histology were less likely to have obstructive sleep apnea, diabetes mellitus or hyperlipidemia and less likely to be on lipid lowering medications.

**Table 1:** Clinical Characteristics of Patients with Normal Histology and NAFLD.

	Normal (n = 49)	N NAFLD (n = 110)	P Value
	n (%)	n (%)	
**Age (years)**	40.3 (10.4)	47.8 (13.6)	0.0007
**Women**	45 (91.8)	87 (79.1)	0.06
**BMI, kg/m^2^ mean(SD)**	45.7 (5.4)	47.5 (8.6)	0.19
**Ethnicity**			
**White**	22 (44.9)	85 (77.3)	
**Black**	27(55.1)	25 (22.7)	<0.0001
**Obstructive Sleep Apnea**	9 (18.0)	65 (59.1)	<0.0001
**Coronary Artery Disease**	9 (18.8)	13 (9.8)	0.10
**Type 2 Diabetes Mellitus**	4 (8.2)	49 (36.3)	0.002
**Lipid Lowering Medication Use**	7 (14.3)	53 (48.2)	<0.0001
**Hypertension**	22 (51.2)	71 (56.4)	0.55
**Hyperlipidemia**	7 (14.3)	56 (44.4)	0.0003
**Tobacco use**	2 (4.1)	7 (6.4)	0.67

**Table 2:** Liver Histology Characteristics of NAFLD Group.

	NAFLD (n = 110)
	n (%)
**Steatosis Grade**	
**0**	12 (10.9)
**1**	61 (55.5)
**2**	25 (22.7)
**3**	12 (10.9)
**Fibrosis Stage**	
**0**	52 (47.3)
**1**	46 (41.8)
**2**	8 (7.3)
**3**	1 (0.9)
**4**	3 (2.7)
**Lobular Inflammation**	
**0**	34 (30.9)
**1**	58 (52.7)
**2**	18 (16.4)
**3**	0 (0)
**Hepatocyte Ballooning**	
**0**	32 (29.1)
**1**	57 (51.8)
**2**	21 (19.1)
**NASH Activity Score**	
**0**–**2**	49 (44.5)
**3**–**4**	33 (30.0)
**5**–**8**	28 (25.5)

### Study Population Metabolic Characteristics (Table 3)

**Table 3:** Metabolic Characteristics of Patients with Normal Histology and NAFLD.

	Normal	NAFLD	P Value
**Mean ALT, U/L (SD)**	16.9 (7.9)	28.3 (27.1)	0.0042
**Mean glucose, mg/dL (SD)**	105.8 (35.3)	126.1 (41.1)	0.004
**Mean Insulin, µU/mL (SD)**	20.8 (15.1)	32.4 (29.0)	0.01
**HOMA-IR (SD)**	2.5 (1.6)	3.5 (1.7)	0.001
**C Reactive Protein (SD)**	1.1 (0.96)	1.0 (0.75)	0.64
**Mean Total Cholesterol, mg/dL**	170.6 (32.7)	161.3 (40.2)	0.16
**Mean HDL, mg/dL**	49.5 (14.5)	42.3 (11.1)	0.009
**Mean LDL, mg/dL**	98.2 (24.3)	94.2 (32.7)	0.45
**Mean Triglycerides, mg/dL**	114.6 (64.8)	133.4 (90.2)	0.19
**Mean Non-HDL Cholesterol, mg/dl**	121.1 (30.4)	119.0 (36.5)	0.73

Patients in the normal group had lower mean ALT, glucose, insulin, and homeostatic model assessment for insulin resistance (HOMA-IR) than those in the NAFLD group. In addition, patients in the normal histology group had higher mean HDL than the NAFLD group (49.5 vs 42.3, p = 0.009) and a higher proportion of patients in the normal group had HDL>50 mg/dL than in the NAFLD group (p = 0.0098). No difference in total cholesterol, LDL, triglycerides, or non-HDL-C was seen between the groups.

### Factors that Predicted Absence of NAFLD

Several previously identified important factors that predicted the absence of NAFLD in obese patients were confirmed. Black race was the strongest predictor of the absence of NAFLD and was associated with an odds ratio (OR) of 6.8 (95% confidence interval [CI] 2.4–18.9) for normal histology. Insulin resistance as measured by fasting insulin, fasting glucose, diagnosis of diabetes mellitus and HOMA-IR were also predictive of normal histology. A low HOMA-IR had the strongest association with the presence of normal histology (OR 1.4, 95% CI 1.03–1.9). This indicates that for each unit decrease in HOMA-IR, the risk of having normal liver histology increases 1.4 fold. In addition, a low HDL was associated with a *decreased* likelihood of normal liver histology (OR 0.38, 95% CI 0.05–0.83). No other lipid values were predictive of normal histology, including non-HDL cholesterol. Finally, a low ALT was associated with an increased prevalence of normal liver histology with an OR of 1.06 (95% CI1.01–1.1).

While black race and a favorable metabolic profile have previously been associated with normal liver histology, we identified the absence of obstructive sleep apnea, as a novel factor associated with normal liver histology. OSA was present in only 9 subjects (18%) with normal liver histology compared to 65 subjects (59.1%) with NAFLD (p = <0.001). The absence of obstructive sleep apnea was also strongly associated with the presence of normal histology, with an OR of 5.6, (95% CI 2.0–16.1). Self-reported use of continuous positive airway pressure (CPAP) for OSA was not significantly different between groups but formal compliance was not assessed.

### Score to Predict Normal Histology

Multivariate regression controlling for BMI found that race, absence of obstructive sleep apnea, HOMA-IR and ALT predicted the presence of normal liver histology with an area under the ROC curve of 0.85. The addition of diabetes mellitus, glucose, insulin, age, gender, the presence of hyperlipidemia or HDL value did not improve this model. Using the clinical and metabolic factors that differed between the normal and NAFLD groups, a weighted score was constructed to predict the presence of normal liver histology.

**Figure 1:**
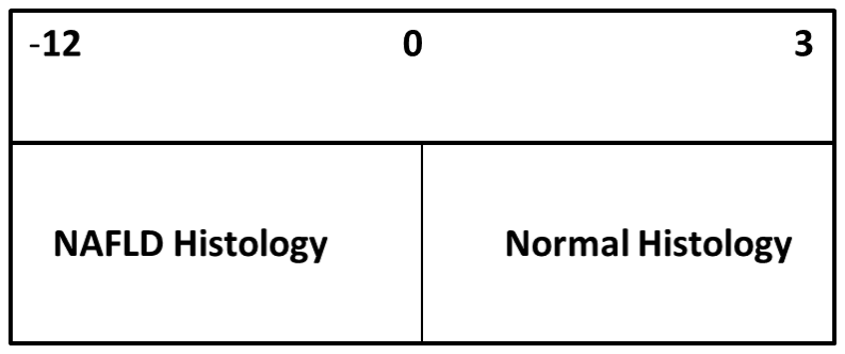
This figure graphically displays the range of the scores for the Healthy Liver Score and the corresponding liver histology.

Our predictive score is: 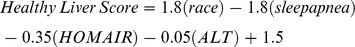



The presence of obstructive sleep apnea is coded as 1 and the absence as value of 0. Black race receives a value of 1 and white patients receive a value of 0. The Healthy Liver Score values range from −12 to 3 in our cohort. (Figure 1).

We evaluated the use of various Healthy Liver Score values to predict normal histology (Figure 1). We found that a score of 0 or greater maximized the combined sensitivity and specificity for predicting normal liver histology with a sensitivity of 59.5% and specificity of 92.7%. While this score has a relatively low sensitivity, the high specificity minimizes the risk of falsely predicting normal liver histology, suggesting its value as a tool to refer patients for NAFLD screening.

We also determined the positive predictive value (PPV) and negative predictive value (NPV) of the Healthy Liver Score. The prevalence of normal liver histology in patients undergoing weight loss surgery varies and can range from 1–15%.^7^Using the extremes of prevalence (1% and 15%), we predicted the relative PPV and NPV. At the extremes of prevalence, our score had a high NPV of 99.2% at 1% prevalence and 92.8% at 15% prevalence. This indicates that a score of “not normal” or less than 0 has a high probability of correctly identifying patients with NAFLD (or those without normal liver histology). The PPV of our score is far less: 14% at 1% prevalence and 56.2% at 15% prevalence of normal histology. Thus, some patients without NAFLD will be placed in the screening group but for a screening score the inclusion of normal patients is preferable to the exclusion of abnormal (or NAFLD) patients.

### Validation Cohort

The performance of the Healthy Liver Score was evaluated in an independent cohort of 92 subjects undergoing weight loss surgery. Twenty-seven subjects had normal liver histology and 65 had NAFLD on liver biopsy. Using a cut-off of > = 0 for normal liver, the Healthy Liver Score had similar performance characteristics with a 44.4% sensitivity and a 96.9% specificity, replicating our findings in the initial cohort.

## Discussion

This study sought to identify factors associated with normal liver histology in patients with obesity. Identification of protective factors can aid in guiding NAFLD screening among high risk patients as well as further the understanding of NAFLD pathogenesis. The inclusion of patients undergoing weight loss surgery offers insight into a unique subset of patients: those with normal liver biopsies that are rarely available. This cohort allows for the identification of protective factors against the development of histologically confirmed NAFLD in an otherwise high risk group.

Our study confirmed several known factors associated with normal liver histology including black race, low HOMA-IR, and low ALT. Uniquely, we found that the absence of sleep apnea was significantly protective against the development of NAFLD, even when controlled for BMI. Studies suggest that obstructive sleep apnea is associated with liver injury.[10] Chronic intermittent hypoxia correlates with NASH activity score and fibrosis.[11] The mechanism for this relationship remains unclear but hypoxia induced by sleep apnea has been shown to increase expression of lipogenic genes and decrease expression of genes regulating mitochondrial beta oxidation in murine models leading to increased hepatic triglyceride storage.[12] In addition, hypoxia has been associated with increased lipid peroxidation and increased pro-inflammatory cytokines including IL-6, chemokine macrophage inflammatory protein-2 and IL-1β, leading to increased hepatic inflammation in the setting of pre-existing steatosis.[13] Our study lends further support to the notion of an important interaction between sleep apnea and NAFLD by demonstrating that the *absence* of sleep apnea may *protect* not only from the development of steatohepatitis but also steatosis itself. Further study is required to determine the impact of sleep apnea on the development of NAFLD and the impact of effective treatment of sleep apnea (CPAP) on NAFLD regression

Using the identified multivariate predictive factors for normal liver histology, we constructed the Healthy Liver Score. This score was strongest when race, sleep apnea, HOMA-IR and ALT were included. The Healthy Liver Score can aid in the identification of high risk patients with obesity most likely to have normal liver histology and exclude these patients from NAFLD screening.

There are several important limitations of our study that will require further evaluation. First, our Healthy Liver Score was derived from a cohort of bariatric surgery patients but before it can be widely used it requires validation in independent surgical cohorts. In addition, our study included only white and black subjects, thus the Healthy Liver Score cannot be applied to other ethnicities. This score should be evaluated more broadly, specifically in Hispanic patients who are at highest risk of NAFLD. In addition, the Healthy Liver Score was derived from a weight loss surgery cohort and may be less applicable to other high risk populations such as patients with diabetes or those referred to specialty liver clinics. Further evaluation of the Healthy Liver Score in broader populations is underway. Second, our study was retrospective in nature and thus the OSA diagnosis was derived from documentation in the medical records of OSA. We were unable to validate whether these patients had formal sleep studies and for those who reported CPAP use, the level of compliance with CPAP. Prospective evaluation is needed to comprehensively diagnosis OSA and monitor compliance. Finally, our study was retrospective in nature and not able to quantify the amount of weight loss patients may have experienced prior to surgery. While varying by surgery, patients are often instructed to lose weight immediately prior to surgery to decrease liver size (so called “shrink the liver” diets) and potentially decrease operative time and complications. This pre-operative weight loss may decrease steatosis and thus impact our findings. Future studies will need to carefully account for any pre-operative weight loss.

In summary, our study identified factors protective against the development of NAFLD in an otherwise high risk population. The identification of protective factors, most importantly the absence of OSA, offers a unique insight into the forces that ameliorate or modulate the driving forces underlying NAFLD. Identification of the mediators of protection, in time could lead to rational approaches to prevention or treatment of patients at high risk of NAFLD.
